# Supplementary material for: Cytomegalovirus Infection Impairs the Mobilization of Tissue-Resident Innate Lymphoid Cells into the Peripheral Blood Compartment in Response to Acute Exercise
Source: Viruses. 2021 Aug 3;13(8):1535. doi: 10.3390/v13081535 (PMC8402764; doi:10.3390/v13081535)
Supplement: Supplementary file 1 [file viruses-13-01535-s001.zip › viruses-1302597-supplementary.pdf]

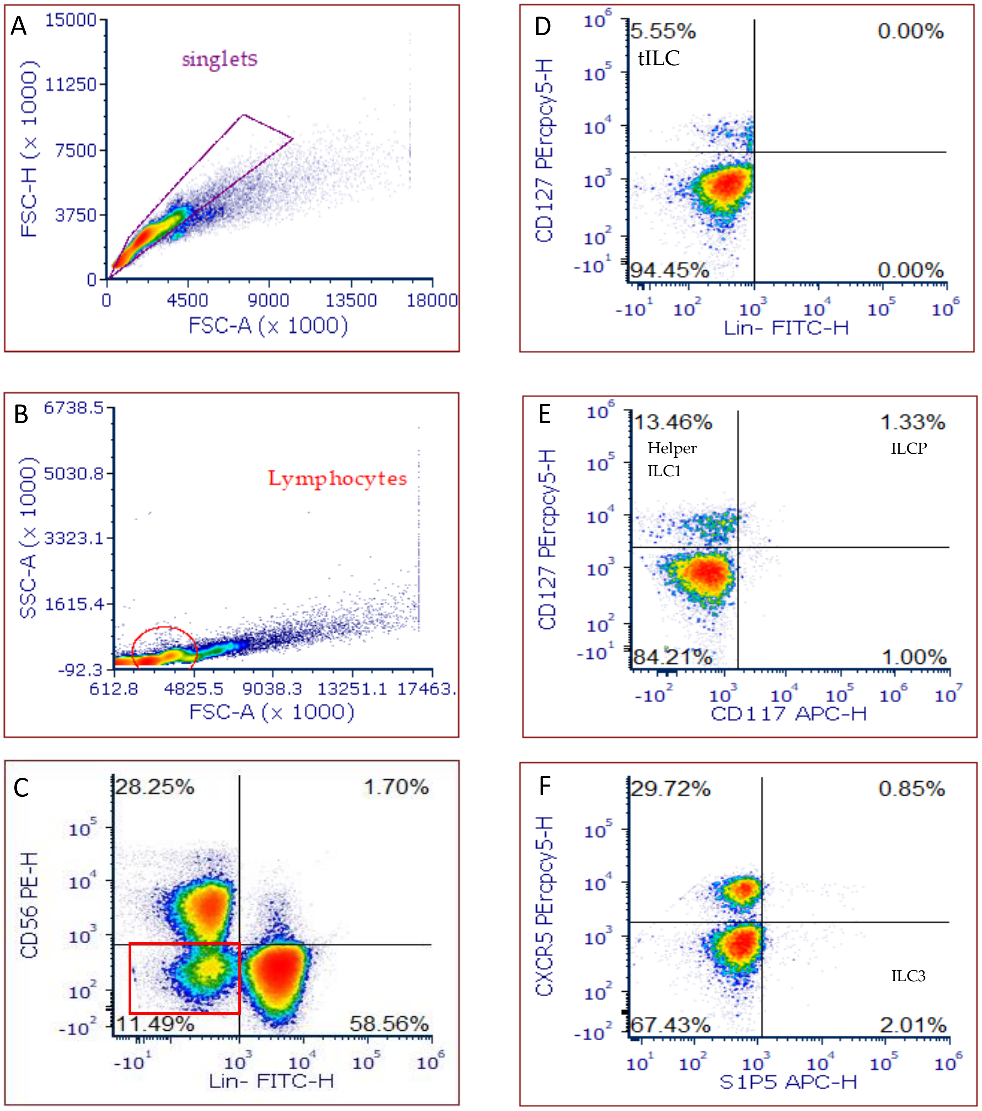

Supplementary Figure S1. The gating strategy for the different Innate Lymphoid Cell (ILC) subsets. Once single cells were identified (A), Peripheral Blood Mononuclear Cells (PBMCs) were gated (B) to isolate non-NK cells (C). Resting and post-exercise total ILC (tILC) (D), Helper ILC1 (E), ILCP (E) and ILC 3 (F) were quantified from this gate. Representative plots are displayed from a post-exercise CMV^{neg} participant.

Supplementary Table S1. The number of NK cell expressing different Chemokine receptors before (pre) and after (post) exercise in all participants (n=22).

Phenotype	NK cell subtype	Pre-Exercise (cell/uL)	Post-Exercise (cell/uL)	p-value
**CX3CR1+/CXCR1-**	NK total	127.33±75.97	405.06±230.34	p<0.001
	$CD56^{dim}$	112.22±71.12	367.18±207.30	p<0.001
	$CD56^{bright}$	6.23±3.0	46.80±15.77	p<0.001
**CX3CR1+/CXCR1+**	NK total	18.98±15.49	79.06±52.90	p<0.001
	$CD56^{dim}$	18.28±17.83	76.59±52.70	p<0.001
	$CD56^{bright}$	0.96±0.89	2.80±2.50	p<0.001
**CX3CR1-/CXCR1+**	NK total	3.97±3.45	5.34±4.02	p<0.05
	$CD56^{dim}$	3.71±3.09	4.06±3.8.3	p>0.05
	$CD56^{bright}$	0.39±0.34	1.00±1.16	p<0.05
**CCR7+/CD62L-**	NK total	3.32±4.13	4.61±4.08	p>0.05
	$CD56^{dim}$	3.35±4.30	4.57±4.11	p>0.05
	$CD56^{bright}$	0.09±0.22	0.15±0.18	p>0.05
**CCR7+/CD62L+**	NK total	6.38±4.22	10.94±6.65	p<0.001
	$CD56^{dim}$	5.25±3.53	9.16±5.81	p<0.001
	$CD56^{bright}$	1.26±1.03	2.04±2.40	p>0.05
**CCR7-/CD62L+**	NK total	73.74±32.30	186.66±87.22	p<0.001
	$CD56^{dim}$	61.61±30.06	161.41±77.48	p<0.001
	$CD56^{bright}$	11.37±5.65	24.01±16.7	p<0.001
**CXCR3+/CCR4-**	NK total	8.45±4.76	21.17±14.94	p<0.001
	$CD56^{dim}$	4.42±2.63	11.37±10.08	p<0.001
	$CD56^{bright}$	3.81±2.86	: 8.51±5.18	p<0.001
**CXCR3+/CCR4+**	NK total	0.42±0.32	1.17±1.18	p<0.01
	$CD56^{dim}$	0.14±0.15	0.52±0.90	p<0.05
	$CD56^{bright}$	0.25±0.24	0.62±0.67	p<0.01
**CXCR3-/CCR4+**	NK total	2.02±1.53	8.38±12.17	p<0.05
	$CD56^{dim}$	1.66±1.41	7.62±11.50	p<0.05
	$CD56^{bright}$	1.41±1.29	0.81±0.83	p<0.01
**CCR6+/CCR4-**	NK total	4.66 2.72	11.32±6.64	p<0.001
	$CD56^{dim}$	1.61±1.16	4.35±4.11	p<0.01
	$CD56^{bright}$	3.43±2.66	7±5.51	p<0.001
**CCR6+/CCR4+**	NK total	0.32±0.29	0.75±0.58	p<0.001
	$CD56^{dim}$	0.09±0.17	0.35±0.58	p<0.05

	$CD56^{bright}$	0.26±0.27	0.42±0.41	p<0.01
**CCR6-/CCR4+**	NK total	2.26±1.63	6.98±9.25	p<0.05
	$CD56^{dim}$	1.77±1.34	6.01±8.40	p<0.05
	$CD56^{bright}$	0.54±0.40	0.97±0.96	p<0.01
**CXCR5+/S1P5-**	NK total	5.09±2.40	12.37±8.42	p<0.001
	$CD56^{dim}$	1.97±1.32	5.87±5.86	p<0.01
	$CD56^{bright}$	3.23±1.83	7.17±4.84	p<0.001
**CXCR5+/S1P5+**	NK total	0.44±0.34	1.31±1.90	p<0.05
	$CD56^{dim}$	0.07±0.08	0.38±1.10	p>0.05
	$CD56^{bright}$	0.39±0.29	1.04±1.12	p<0.01
**CXCR5-/S1P5+**	NK total	2.43±1.34	7.12±9.76	p<0.05
	$CD56^{dim}$	1.69±1.04	5.50±8.92	p<0.05
	$CD56^{bright}$	9.24±5.15	25.79±13.46	p<0.001
**CCR7+/CXCR4-**	NK total	4.45±1.53	10.72±4.88	p<0.001
	$CD56^{dim}$	1.92±1.08	4.89±3.50	p<0.001
	$CD56^{bright}$	2.57±1.49	5.72±3.52	p<0.001
**CCR7+/CXCR4+**	NK total	1.66±1.4	3.16±3.38	p<0.05
	$CD56^{dim}$	1.55±1.36	2.78±3.16	p<0.05
	$CD56^{bright}$	0.15±0.14	0.25±0.35	p>0.05
**CCR7-/CXCR4+**	NK total	3.76±2.85	7.68±3.89	p<0.001
	$CD56^{dim}$	3.33±2.86	6.96±3.48	p<0.001
	$CD56^{bright}$	0.37±0.31	0.52±0.45	p>0.05
**CXCR6+/LFA-1-**	NK total	4.36±2.56	11.78±6.56	p<0.001
	$CD56^{dim}$	2.78±1.81	7.79±5.11	p<0.001
	$CD56^{bright}$	1.77±1.29	3.70±2.13	p<0.001
**CXCR6+/LFA-1+**	NK total	0.87±0.79	2.16±2.40	p<0.01
	$CD56^{dim}$	0.65±0.52	1.66±1.59	p<0.001
	$CD56^{bright}$	0.11±0.10	0.35±0.50	p<0.01
**CXCR6-/LFA-1+**	NK total	1.22±1.35	3.75±4.09	p<0.05
	$CD56^{dim}$	1.00±1.30	3.23±4.16	p<0.05
	$CD56^{bright}$	0.25±0.20	0.45±0.41	p<0.05
